# Genetic determinants of antithyroid drug-induced agranulocytosis by human leukocyte antigen genotyping and genome-wide association study

**DOI:** 10.1038/ncomms8633

**Published:** 2015-07-07

**Authors:** Pei-Lung Chen, Shyang-Rong Shih, Pei-Wen Wang, Ying-Chao Lin, Chen-Chung Chu, Jung-Hsin Lin, Szu-Chi Chen, Ching-Chung Chang, Tien-Shang Huang, Keh Sung Tsai, Fen-Yu Tseng, Chih-Yuan Wang, Jin-Ying Lu, Wei-Yih Chiu, Chien-Ching Chang, Yu-Hsuan Chen, Yuan-Tsong Chen, Cathy Shen-Jang Fann, Wei-Shiung Yang, Tien-Chun Chang

**Affiliations:** 1 Division of Endocrinology and Metabolism, Department of Internal Medicine, National Taiwan University Hospital, Taipei 100, Taiwan.; 2 Department of Medical Genetics, National Taiwan University Hospital, Taipei 100, Taiwan.; 3 Graduate Institute of Medical Genomics and Proteomics, College of Medicine, National Taiwan University, Taipei 100, Taiwan.; 4 Graduate Institute of Clinical Medicine, College of Medicine, National Taiwan University, Taipei 100, Taiwan.; 5 Research Center for Developmental Biology and Regenerative Medicine, National Taiwan University, Taipei 106, Taiwan.; 6 Department of Medicine, College of Medicine, National Taiwan University, Taipei 100, Taiwan.; 7 Department of Internal Medicine, Kaohsiung Chang Gung Memorial Hospital and Chang Gung University College of Medicine, Kaohsiung 833, Taiwan.; 8 Institute of Biomedical Sciences, Academia Sinica, Taipei 115, Taiwan.; 9 Immunogenetics Laboratory, Department of Medical Research, Mackay Memorial Hospital, Taipei 251, Taiwan.; 10 School of Pharmacy, National Taiwan University, Taipei 100, Taiwan.; 11 Research Center for Applied Sciences, Academia Sinica, Taipei 833, Taiwan.; 12 Department of Internal Medicine, New Taipei City Hospital, New Taipei 220, Taiwan.; 13 Department of Internal Medicine, China Medical University Hospital, Taichung 404, Taiwan.; 14 Department of Internal Medicine, China Medical University, Taichung 404, Taiwan.; 15 Department of Social Medicine, College of Medicine, National Taiwan University, Taipei 100, Taiwan.; 16 Department of Internal Medicine, Cathay General Hospital, Taipei 106, Taiwan.; 17 Department of Laboratory Medicine, National Taiwan University Hospital, Taipei 100, Taiwan.; 18 Department of Laboratory Medicine, College of Medicine, National Taiwan University, Taipei 100, Taiwan.; 19 Department of Pediatrics, Duke University Medical Center, Durham, North Carolina 27708, USA.

## Abstract

Graves' disease is the leading cause of hyperthyroidism affecting 1.0–1.6% of the population. Antithyroid drugs are the treatment cornerstone, but may cause life-threatening agranulocytosis. Here we conduct a two-stage association study on two separate subject sets (in total 42 agranulocytosis cases and 1,208 Graves' disease controls), using direct human leukocyte antigen genotyping and SNP-based genome-wide association study. We demonstrate HLA-B*38:02 (Armitage trend *P*_combined_=6.75 × 10^−32^) and HLA-DRB1*08:03 (*P*_combined_=1.83 × 10^−9^) as independent susceptibility loci. The genome-wide association study identifies the same signals. Estimated odds ratios for these two loci comparing effective allele carriers to non-carriers are 21.48 (95% confidence interval=11.13–41.48) and 6.13 (95% confidence interval=3.28–11.46), respectively. Carrying both HLA-B*38:02 and HLA-DRB1*08:03 increases odds ratio to 48.41 (*P*_combined_=3.32 × 10^−21^, 95% confidence interval=21.66–108.22). Our results could be useful for antithyroid-induced agranulocytosis and potentially for agranulocytosis caused by other chemicals.

## Abstract (web summary)

Graves' disease is the leading cause of hyperthyroidism but treatment options can cause life-threatening complications. Chen *et al*. conduct two-stage direct HLA genotyping and genome-wide association studies to identify HLA-B*38:02 and HLA-DRB1*08:03 as major pharmacogenetic determinants.

## Graphical abstract

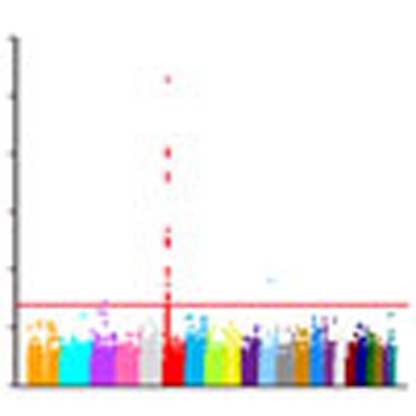

Graves' Disease (GD, MIM 27500) is the leading cause of hyperthyroidism, manifested with diffuse goitre, thyroid hyperfunction, thyroid-specific auto-antibodies, ophthalmopathy and/or dermopathy^1^. Its prevalence in the general population is as high as 1.0–1.6%, more common in females^2^,^3^. Antithyroid drugs (ATDs, including methimazole, carbimazole and propylthiouracil) are relatively simple molecules known as thionamides^4^, which have been cornerstones of GD treatment across the globe^4^. ATD-induced agranulocytosis, namely thionamide-induced agranulocytosis (TiA, defined as an absolute granulocyte count <500 mm^−3^ while taking ATDs), is the most feared adverse effect of ATDs^4^ and can occur in 0.1–0.37% of GD patients receiving these medications^5^,^6^. Agranulocytosis is life-threatening and can be induced by a variety of non-chemotherapy drugs^7^,^8^. Among the 11 most common offending drugs summarized in a recent review^7^, ATDs accounted for 3 of the 11, and the other drugs included clozapine, dapsone, dipyrone, penicillin G, procainamide, rituximab, sulfasalazine and ticlopidine^7^. Drug-induced adverse effects could be broadly categorized into several types^9^,^10^, including type A (dose-related, augmented) and type B (non-dose-related, bizarre), and can have different genetic predisposition^11^. TiA belongs to type B. Overall, pharmacogenetic studies related to drug-induced agranulocytosis were scanty and very often were inconclusive^12^,^13^,^14^,^15^,^16^. On the other hand, non-genetic risk factors remain elusive as well. Human leukocyte antigen (HLA) genes have been associated with many drug-induced adverse effects, including carbamazepine-induced Stevens–Johnson syndrome (HLA-B*15:02 and HLA-A*31:01)^17^,^18^, abacavir-induced hypersensitivity syndrome (HLA-B*57:01)^19^,^20^, lapatinib-induced liver injury (HLA-DQA1*02:01)^21^ and so on. Non-HLA genes also cause numerous drug-induced adverse effects through various pharmacokinetic and pharmacodynamic mechanisms^11^. To comprehensively identify HLA and non-HLA genetic susceptibility to TiA, we conducted both direct HLA genotyping and genome-wide association study (GWAS) on GD patients with or without TiA. We demonstrate HLA-B*38:02 and HLA-DRB1*08:03 as independent, major susceptibility loci. Importantly, the GWAS data identify the same HLA signals, as well as one association peak at chromosome 3q13 with borderline significance. Three-dimensional (3D) structure modelling of the two HLA proteins and ATDs provides possible binding modes.

## Results

### Direct classical HLA loci genotyping and association tests

**Figure 1:**
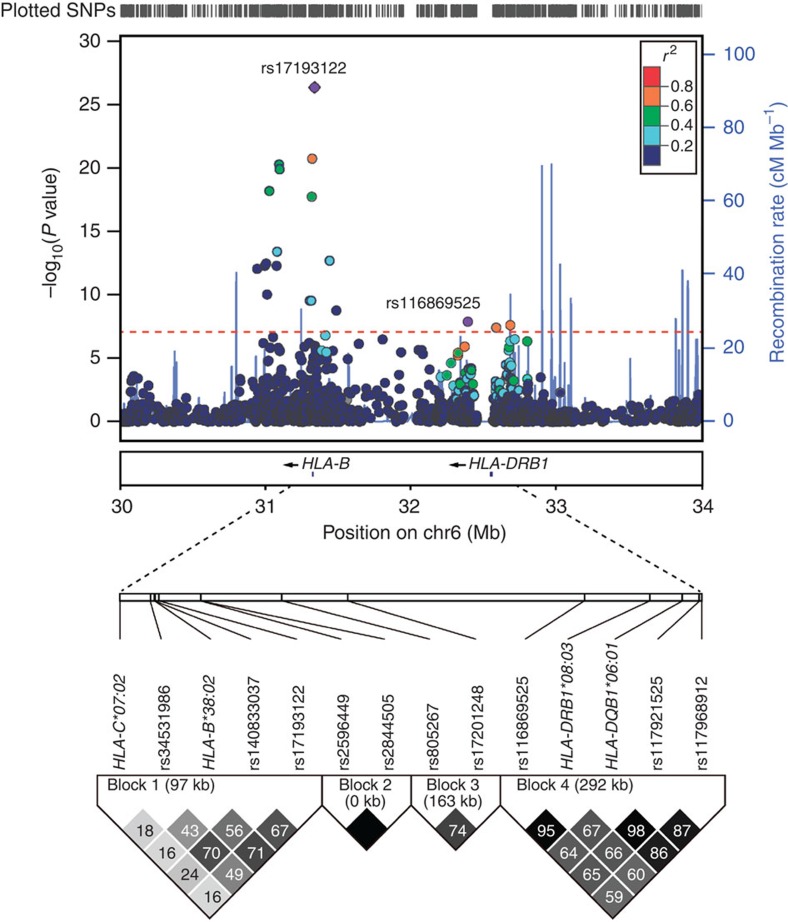
Regional plot of association signals and LD patterns at the HLA region. (Top panel): The *x* axis shows chromosomal positions. The left *y* axis shows –log_10_*P* values from the GWAS. Colours of the dots indicate the LD relationship between rs17193122 or rs116869525 and their neighbouring SNPs in the 4-Mb region (30–32 Mb for rs17193122 and 32–34 Mb for rs116869525) based on our own data (969 samples). The red dashed line indicates the significance threshold (9.56 × 10^−8^). The right *y* axis shows the recombination rate between SNPs calculated based on Asian-ancestry population data from the 1000 Genomes Project. (Bottom panel): LD map based on *r*^2^ in the associated regions using genotype results from our own data (969 samples). We construct the plot using the Haploview software version 4.2, and *r*^2^ (× 100) values are depicted in the diamonds.

The structure of the study and the number of participants are summarized in Supplementary Table 1. For HLA association study using six directly genotyped classical HLA loci (*HLA-A*, *-B*, *-C*, *-DPB1*, *-DQB1* and *-DRB1*), we observed two significant associations at HLA-B*38:02 (*P*=1.59 × 10^−9^, note: *P* values are Armitage trend *P* values in this manuscript, if not otherwise specified) and HLA-DRB1*08:03 (*P*=1.24 × 10^−5^) in the first-stage study (21 TiA cases versus 662 GD controls). The second-stage study (additional 21 TiA cases versus 546 GD controls) replicated the two associations at HLA-B*38:02 (*P*=1.59 × 10^−27^) and HLA-DRB1*08:03 (*P*=3.91 × 10^−5^). Combined results from 42 TiA cases and 1,208 GD controls showed even stronger associations: HLA-B*38:02 (*P*_combined_=6.75 × 10^−32^) and HLA-DRB1*08:03 (*P*_combined_=1.83 × 10^−9^, Table 1). The allele frequencies of HLA-B*38:02 were 29.8% in the TiA cases, 3.3% in the GD controls and 3.3% in general population in Taiwan^22^. The allele frequencies of HLA-DRB1*08:03 were 28.6% in the TiA cases, 8.4% in the GD controls and 8.6% in general population^22^. The genotypic information for 42 TiA cases is shown in Supplementary Table 2. The allele frequencies of 6 classical HLA genes in 42 TiA cases and in 1,208 GD controls can be found in Supplementary Data 1. Odds ratios of these two HLA alleles for effective allele carriers compared with non-carriers were 21.48 (95% confidence interval, CI=11.13–41.48) and 6.13 (95% CI=3.28–11.46), respectively. These two loci are not in the same linkage disequilibrium (LD) block (Fig. 1 and Supplementary Fig. 1). In addition, HLA-A*02:03, HLA-C*07:02 and HLA-DQB1*06:01 also showed significant association signals (*P*=4.22 × 10^−8^, 4.09 × 10^−6^ and 1.29 × 10^−6^, respectively) but were found to be in LD with either HLA-B*38:02 or HLA-DRB1*08:03 (Fig. 1 and Supplementary Fig. 1), therefore not considered as independent associations. HLA imputation using single-nucleotide polymorphisms (SNPs) at the HLA region was also carried out. The results were highly similar to those based on direct HLA typing (Table 2 and Supplementary Table 3).

### Genome-wide association study

**Figure 2:**
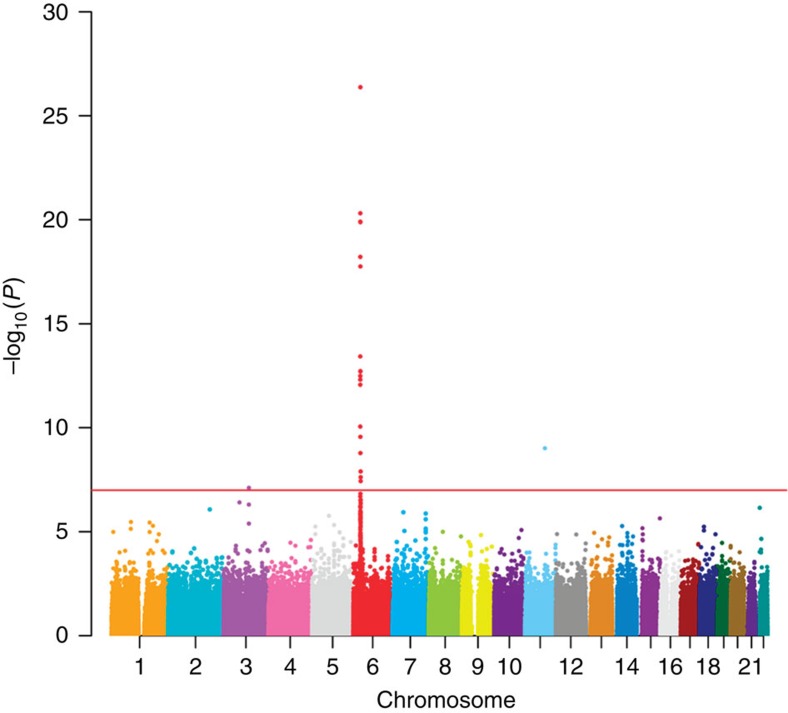
Association results from the genome-wide scan. Manhattan plot shows the genome-wide *P* values (−log_10_*P* value) of the Cochran–Armitage test for trend from 522,980 polymorphic SNPs in 42 TiA cases and 927 GD controls. SNPs on each chromosome were given the same colour. Red line, *P*=9.56 × 10^−8^ (as the genome-wide significance cutoff value of this study).

GWAS using Affymetrix Axiom Genome-Wide CHB 1 SNP Array (a total of 522,980 autosomal SNPs after quality-control process) showed an excess of small *P* values at the tail of the quantile–quantile distribution plot (Supplementary Fig. 2) but only limited evidence of inflation caused by population stratification (genomic inflation factor *λ*_GC_=1.01). The multidimensional scaling (MDS) and principal component analyses showed no outliers (Supplementary Figs 3 and 4). We identified two independent association signals at the HLA region (Table 1, Figs 1 and 2, Supplementary Table 4). The first signal was represented by rs17193122 (*P*_first stage_=8.18 × 10^−8^, *P*_second stage_=1.15 × 10^−24^, *P*_combined_=4.29 × 10^−27^). The second association signal was represented by rs116869525 (*P*_first stage_=3.88 × 10^−5^, *P*_second stage_=8.24 × 10^−5^, *P*_combined_=1.27 × 10^−8^). In addition to the HLA region at chromosome 6p21, the results also revealed one non-HLA locus showing borderline GWAS association signals, represented by rs56343172 at chromosome 3q13 (*P*_first stage_=1.71 × 10^−5^, *P*_second stage_=7.32 × 10^−4^, *P*_combined_=7.75 × 10^−8^).

### HLA-B*38:02 and HLA-DRB1*08:03 have independent effects

The associations at the HLA region derived from two different genotyping techniques (direct classical HLA genotyping and Affymetrix SNP Array) provided convergent evidence of the same signals. The first group of SNPs, represented by rs17193122, were in LD with HLA-B*38:02 (Fig. 1), and their associations with TiA disappeared when they were tested in the regression model containing HLA-B*38:02. Similarly, the second group of SNPs, represented by rs116869525, were in LD with HLA-DRB1*08:03 (Fig. 1), and did not show significant association as well in the regression model containing HLA-DRB1*08:03.

Although HLA-B*38:02 and HLA-DRB1*08:03 are sometimes in the same extended HLA haplotype, we could demonstrate their independent effects on the basis of three lines of evidence. First, the LD block patterns of TiA cases and GD controls (Fig. 1 and Supplementary Fig. 1) clearly showed that these two alleles resided in two different LD blocks. Second, in our stepwise regression model, these two alleles demonstrated independent effects (*P*=1.89 × 10^−16^ for HLA-B*38:02 and *P*=2.24 × 10^−6^ for HLA-DRB1*08:03, respectively, Supplementary Table 5). Third, in those individuals who carried HLA-B*38:02 (25 in TiA cases and 77 in GD controls), the percentage of individuals who also carried HLA-DRB1*08:03 was statistically higher in TiA cases (16/25=64%) than in GD controls (15/76=19.74%, *P*=9.44 × 10^−5^).

### HLA-B*38:02 and HLA-DRB1*08:03 have major effects

We observed none of the 42 TiA cases to be homozygous for HLA-B*38:02, and only 2 of them to be homozygous for HLA-DRB1*08:03. Following the traditional practice for assessing the risk of HLA-related drug adverse effect^17^,^23^, we calculated disease odds ratio for these loci according to the effective allele carrier status. HLA-B*38:02 was present in 59.52% of TiA cases, but only in 6.41% of GD controls, which transformed into an odds ratio of 21.48 (*P*=6.28 × 10^−18^, 95% CI=11.13–41.48). HLA-DRB1*08:03 was present in 52.38% of TiA cases, but only in 15.22% of GD controls, which transformed into an odds ratio of 6.13 (*P*=1.35 × 10^−8^, 95% CI=3.28–11.46). For those who carried both HLA-B*38:02 and HLA-DRB1*08:03 (38.10% in TiA cases and 1.26% in GD controls), the odds ratio increased to 48.41 (*P*=3.32 × 10^−21^, 95% CI=21.66–108.22). Using a logistic regression procedure (Supplementary Table 5), the final logistic regression model was:

logit(π)=−4.4936+2.6382 × HLA-B*38:02+1.2857 × HLA-DRB1*08:03 (1)

(both loci under additive model), where π was the probability of TiA.

**Figure 3:**
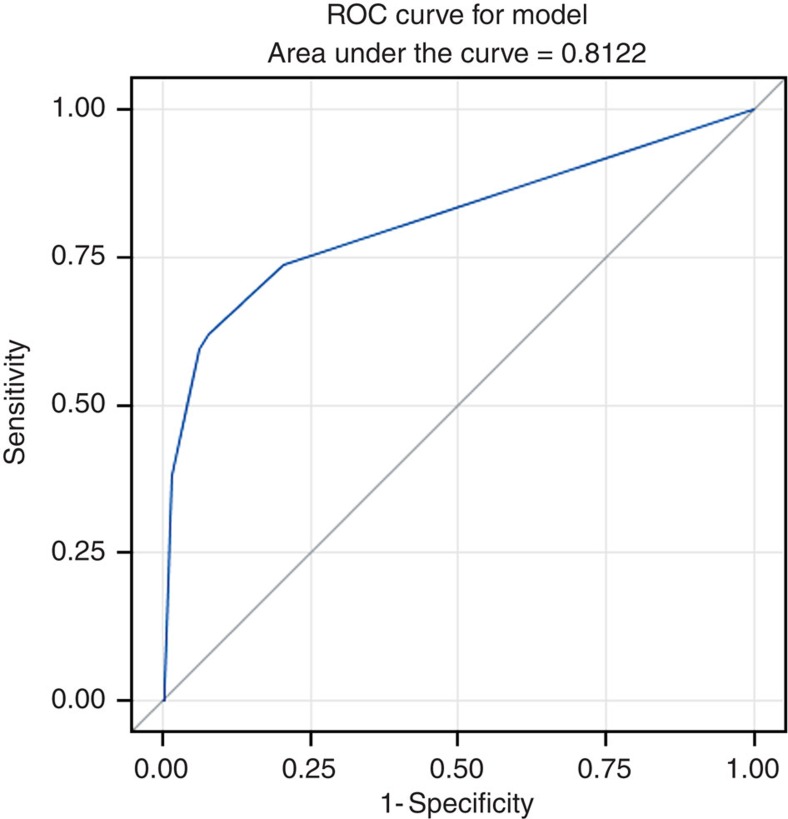
The ROC curve to discriminate TiA cases (*n*=42) from GD controls (*n*=927). Sensitivity, specificity, positive and negative predictive values of the model were 61.09% (95% CI=45.64–76.42), 92.13% (95% CI=90.46–93.60), 21.67% (95% CI=14.67–30.11) and 98.57% (95% CI=97.68–99.18), respectively.

Under this model, the AUC of the receiver operating characteristic (ROC) curve was 81.22% (Fig. 3). Since ∼74% of the TiA patients carry the risk genotypes and routine follow-up of blood cell count is controversial^4^,^5^,^6^. Different follow-up protocols can be proposed on the basis of genetic risk stratification to possibly prevent 74% of this rare condition from occurrence. Further replication is needed to confirm the estimate. Cost-effectiveness analysis will be carried out in further study.

### 3D structure modelling

**Figure 4:**
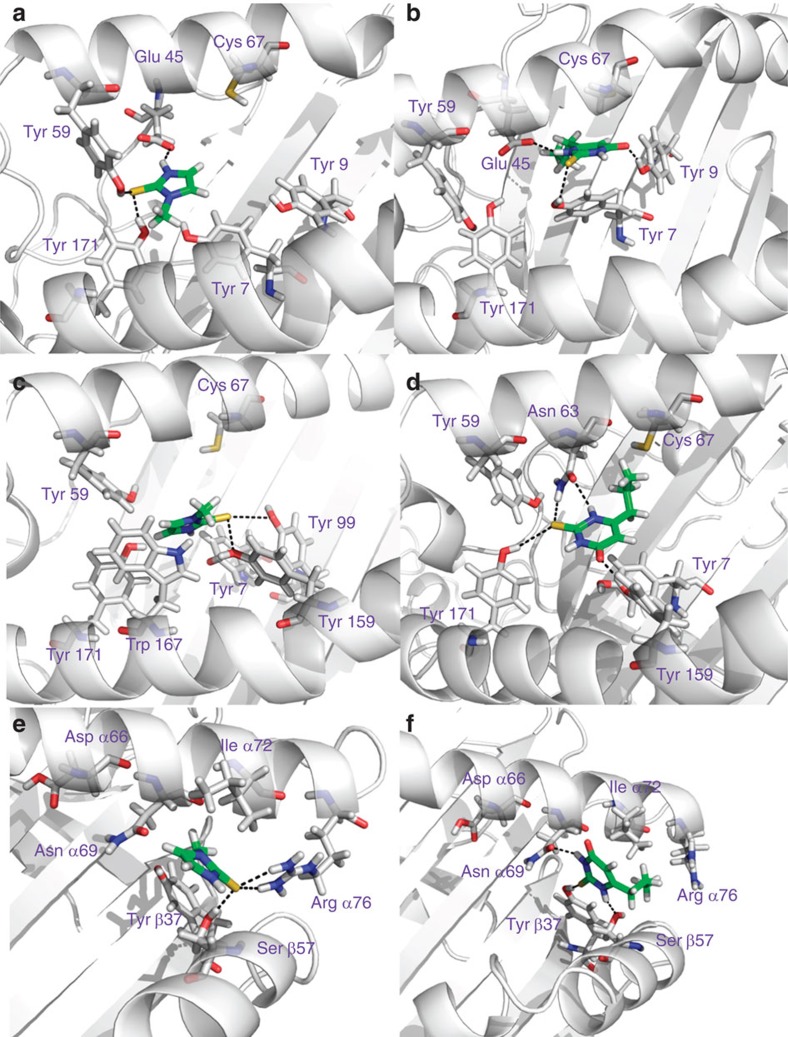
Specific protein–ligand interactions between thionamide drugs and the HLA proteins identified in this work. (**a**) Methimazole-HLA-B*38:02. (**b**) Propylthiouracil-HLA-B*38:02. (**c**) Methimazole-HLA-B*38:01. (**d**) Propylthiouracil-HLA-B*38:01. (**e**) Methimazole-HLA-DRA/DRB1*08:03. (**f**) Propylthiouracil-HLA-DRA/DRB1*08:03 complexes. The predicted binding affinities are within the range between −4.48 and −6.36 kcal mol^−1^.

**Figure 5:**
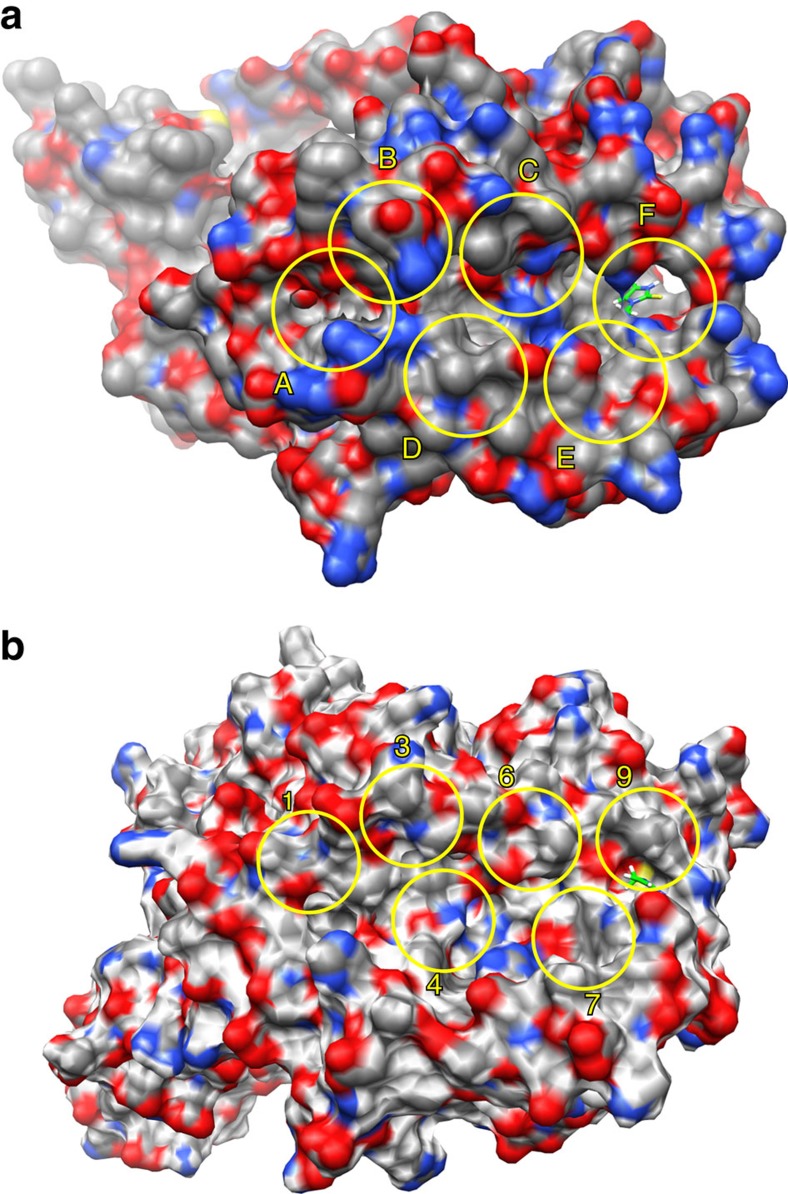
Surface representation of HLA proteins and their sub-pockets. (**a**) HLA-B*38:02. The pocket positions are defined on the basis of the crystal structures of peptide-HLA-B*15:01 complex. The sub-pockets of HLA-B*38:01 are located at similar positions. (**b**) HLA-DRA/DRB1*08:03. The pocket positions are defined on the basis of the crystal structure of peptide-HLA-DR1 complex.

Despite the strong genetic associations, the mechanisms of actions remain enigmatic. To further probe the direct interactions between the HLA proteins and the thionamide drugs, 3D structure modelling, molecular docking and molecular dynamics simulations were carried out to determine the possible binding modes and to estimate the binding affinities of the thionamide–HLA complexes. For HLA-B*38:02 and HLA-B*38:01 proteins, binding modes in pocket B and pocket F exhibits comparable binding affinities in the range between −4.48 and −6.36 kcal mol^−1^. It should be noted that the binding of the thionamide drugs could be further stabilized by the peptide to be presented. Examples of specific protein–ligand interactions stabilizing the thionamide–HLA complexes of binding modes with relatively strong affinities are illustrated in Fig. 4. In the molecular dynamics simulations, it was observed that the thionamide drugs drift and anchor to different sub-pockets of the peptide-binding groove with alternating hydrogen bonds and specific contacts. Cys67 of HLA-B*38:02 and HLA-B*38:01, which signifies the polymorphism of these two HLAs, is found to locate in the neighbourhood of the drug-binding sites. Therefore, it is anticipated that the sulfhydryl groups of the Cys67 and thionamide drugs may form disulfide bonds under the oxidative condition and thereby further stabilize the drug–HLA complex. However, we also note that one of the major binding poses of methimazole at the pocket B of HLA-B*38:01 would cause steric hindrance for the peptide binding. In pocket F of HLA-B*38:02 and HLA-B*38:01 (Fig. 5), and pocket 9 of HLA-DRA/DRB1*08:03 (Fig. 4e,f), one asparagine residue (Asn77 for HLA-B*38:02 and HLA-B*38:01, and Asnα69 for HLA-DRA/DRB1*08:03) was found to play a critical role in stabilizing the drug molecules at the edge of peptide-binding groove, by bonding with the sulfur atom or the nitrogen atom (sometimes both) of the drugs. Besides, Thr80 and Tyr123 in HLA-B*38:02, Tyr74 in HLA-B*38:01, Argα76, Tyrβ37 and Serβ57 in HLA-DRA/DRB1*08:03 provide additional interactions to stabilize the drugs in the F pocket. The peptide-binding groove of HLA-DRA/DRB1*08:03 are rich in aromatic residues and polar residues, which could also trap the thionamide drugs in the binding groove. It should be cautiously noted that here we only depict the most frequently occurring binding modes of many possible ones in the dynamics simulations. Since we did not known which peptides would participate in this process, the binding affinity and the binding modes of the drug in the HLA–peptide complex and the determinants of TiA response were not solved at this moment. Further experiments are needed.

## Discussion

In this study, we performed concurrent direct HLA genotyping and GWAS in a cohort of 42 TiA cases and 1,208 GD controls in a two-stage study, and identified two HLA loci as major genetic determinants of ATD-induced agranulocytosis. This approach had the advantage of exploring the entire genome without bias and also to use two independent genotyping methods to evaluate the effects of classical HLA genes and other non-HLA genes at the HLA region.

For the HLA associations, we demonstrated that HLA-B*38:02 and HLA-DRB1*08:03 had independent major effects. Class I and class II HLA genes have different structures, cell-type distributions and functional roles in the immune system^24^. Therefore, the genetic susceptibility from both classes for a phenotype is quite intriguing. Before our finding, Lucena *et al*.^25^ already reported the association of class I and class II HLA genotypes with amoxicillin–clavulanate-induced liver injury. How both class I and class II HLA genes confer genetic susceptibility to the same drug adverse effect deserves further pathophysiological investigation^23^.

Several additional analyses were performed intending to relate the genotypes of the TiA cases to other characteristics (Supplementary Tables 6–8). Some interesting signals were picked up, such as age at agranulocytosis (Supplementary Table 6) and ATDs administered (Supplementary Table 8). Because the sample size was small for each genotype, it may be premature to draw any conclusion from these.

In genetic studies, the HLA region is challenging to get clear-cut results because of high gene density^24^, enormous polymorphism^26^ and extended LD^24^. Although we demonstrated independent associations between TiA and two classical HLA genes/alleles, namely HLA-B*38:02 and HLA-DRB1*08:03, an association study could not exclude the possibility of nearby non-HLA gene(s) to be the genuine causative gene(s).

Previously, Tamai *et al*.^13^ reported an association of TiA with HLA-DRB1*08032 (the same allele as HLA-DRB1*08:03) in 1996. It is noteworthy that Japanese population has very low frequency (<0.5%) of HLA-B*38:02 (ref. 27); therefore, the results from Tamai *et al*.^13^ further provide evidence supporting the independent effects of HLA-DRB1*08:03 and HLA-B*38:02.

Among all genetic studies of drug-induced agranulocytosis, clozapine-induced agranulocytosis is the most studied^15^,^16^,^23^. HLA-B*38 was associated with clozapine-induced agranulocytosis in Jews, but not in other populations^15^,^16^,^23^,^28^. HLA-B*38 alleles in Caucasians are mainly HLA-B*38:01, not HLA-B*38:02 popular in Asians^27^. The two alleles differ by one amino acid at the 80th position (Supplementary Fig. 5) located at peptide-binding groove pocket F^29^. It is intriguing that two related HLA alleles were associated with agranulocytosis induced by two structurally unrelated drugs.

A couple of caveats need to be addressed. First, the total number of TiA cases in this report was not big. Second, for any single genetic association study, the possibility of population substructure cannot be completely excluded. Although this report is by far the largest TiA genetic study, with the two-stage study design and extensive quality-control steps supporting the validity of the results, it is still crucial to have independent replications in the same and/or other populations in the future.

The susceptibility HLA alleles we discovered (HLA-B*38:02 and HLA-DRB1*08:03) have higher allele frequencies in Asians (0.036 and 0.048, respectively) than in Caucasians (0.004 and 0.002, respectively)^27^. However, the incidence of TiA, according to available surveys, seems to be similar across the globe^4^,^5^,^6^,^30^,^31^. Therefore, these two alleles cannot be the only reason for TiA susceptibility. There are several possible explanations supported by examples from other diseases. For example, the major susceptibility HLA alleles in different populations might be structurally similar alleles, such as different *HLA-DR4* alleles (HLA-DRB1*04:01 and HLA-DRB1*04:05) for type 1 diabetes susceptibility^32^,^33^. Under this hypothesis, HLA-B*38:01 and HLA-DRB1*08:01 might be good TiA candidates in Caucasians. On the other hand, other reasonable hypotheses exist, including different major HLA genes/alleles (such as in GD^22^ and in carbamazepine-induced Stevens–Johnson syndrome^23^), a spectrum of HLA genes/alleles sharing critical HLA amino-acid polymorphism (such as in rheumatoid arthritis^34^), or even non-genetic causes.

## Methods

### Study individuals

GD was diagnosed on the basis of the presence of clinical and biochemical hyperthyroidism together with either the presence of thyroid eye disease or a diffuse goitre and a significant titre of autoantibodies^22^. Enrollment of GD individuals has been a continuous process at the National Taiwan University Hospital (NTUH) starting in August 2001. ATD-induced agranulocytosis, TiA, was initially identified according to the questionnaire from the GD participants at NTUH, but has later been actively searched for from the medical records at NTUH and another tertiary medical centre, Kaohsiung Chang Gung Memorial Hospital (KCGMH). Medical records were reviewed and the patients were excluded if they received other medications that could possibly explain their white blood count drops, such as chemotherapy agents, clozapine, dapsone, dipyrone, penicillin G, procainamide, sulfasalazine or ticlopidine, and so on. Although TiA was classically defined as absolute granulocyte counts <500 mm^−3^ in GD patients taking any of the three thionamides (methimazole, carbimazole and propylthiouracil), it should be noted that these cases were identified by clinical infection, not just by laboratory data. All the patients in this study presented with fever and sore throat with or without skin rash were hospitalized for management. Agranulocytosis developed approximately within 3 months after the start of ATDs. Available medical information of those 42 patients might not be sufficient to completely exclude concurrent viral infection. Causality assessment using widely adapted tools, such as the Naranjo algorithm^35^,^36^ and the World Health Organization Collaborating Centre for International Drug Monitoring, the Uppsala Monitoring Centre (WHO-UMC) causality categories^35^, scored ‘probable' adverse drug reaction for all the 42 cases; the causality assessment did not reach the highest definite/certain category mostly because that re-challenge test was not performed and it was not possible to completely exclude all other possible causes.

For TiA ‘cases', we enrolled 21 patients at NTUH between 2001 and March 2012 and genotyped in our first-stage study (Supplementary Table 1). We used GD patients without agranulocytosis as the controls (GD controls). Our first-stage study comprised 497 unrelated GD patients and the 165 probands from unrelated GD families (only one GD individual from each family) enrolled between 2001 and 2006 (Supplementary Table 1), who were essentially the same individuals described in our previous publication^22^. With initial encouraging positive signals in the first-stage study, we then actively recruited additional 21 TiA patients from NTUH and KCGMH for the second-stage study between March 2013 and March 2014 (Supplementary Table 1). Additional 546 unrelated GD patients enrolled between 2001 and 2011 who were not previously genotyped were then included as the GD controls in the second-stage study (Supplementary Table 1). Two of the 21 first-stage TiA cases and all 662 first-stage GD controls overlapped with the individuals reported in our previous paper^22^. All the other TiA cases and the second-stage GD controls were new participants not previously reported. Power calculations were performed with various MAF and OR settings using QUANTO^37^ (http://biostats.usc.edu/Quanto.html). On the basis of the given sample size of the current GWAS study (TiA case number=42, GD control number=927), the power was reasonably adequate to detect major susceptibility gene(s) (Supplementary Fig. 6). The study was approved by the Institutional Review Board of both NTUH and KCGMH. Written informed consent was obtained from all participants.

### Direct HLA genotyping

There were four kinds of HLA genotyping methods used in this study including (1) Dynal RELI SSO typing kits (Dynal biotech Ltd, Bromborough, Wirral, UK, now part of Life Technologies, Carlsbad, CA, USA, http://www.invitrogen.com/), (2) Gold SSP HLA-DPB1 High-resolution Kit (Invitrogen Corp., now part of Life Technologies http://www.invitrogen.com/), (3) LABType SSO kit (One lambda Inc., Canoga Park, CA, USA, http://www.onelambda.com/) and (4) SeCore HLA Sequence-Based Typing Kit (Life Technologies Corporation, Brown Deer, WI, USA, http://www.lifetechnologies.com/). The first three methods were applied to 2 out of our 21 first-stage TiA cases and all of our 662 first-stage GD controls. The fourth method (SeCore HLA sequence-based typing) was applied to all (including both first-stage and second-stage) of our 42 TiA cases and all of our 546-s-stage GD controls. In addition to the two first-stage TiA cases that were genotyped using more than one genotyping methods, we also randomly selected 24 first-stage GD controls to receive SeCore HLA sequence-based typing, in order to make sure that the genotyping results were consistent across all the genotyping methods.

Dynal RELI SSO genotyping was performed according to the manufacturer's instructions. Briefly, PCR using locus-specific primer sets were applied to amplify both exon 2 and exon 3 of class I (*HLA-A*, *-B* and *-C*) genes or exon 2 of class II *(-DQB1* and *-DRB1*) genes. Subsequently, PCR products were hybridized with sequence-specific oligonucleotide (SSO) probes previously fixed in a linear array on a nylon membrane (*HLA-A*: 48 probes, *-B*: 61 probes, *-C*: 37 probes, *-DQB1*: 41 probes and *-DRB1*: 60 probes). We then interpreted the genotypes using the Pattern Matching programme (Dynal biotech Ltd).

Owing to the lack of the *DPB1* genotyping kit in the Dynal RELI SSO system, we genotyped *HLA-DPB1* based on a sequence-specific primer (SSP) amplification method using ‘Gold SSP *HLA-DPB1* High resolution Kit' according to the manufacturer's protocol. Briefly, 48 PCR reactions were performed for each DNA sample. After PCR amplification and electrophoresis, the patterns of positive amplifications were used to interpret *HLA-DPB1* genotypes with the company's UniMatch software (Invitrogen Corp.).

LABType SSO kit-based genotyping was performed according to the manufacturer's protocol. Briefly, PCR products were hybridized with probes bound to fluorescently coded microspheres (*HLA-A*: 58/61/63 probes, *-B*: 100 probes, *-C*: 56 probes, *-DPB1*: 40 probes, *-DQB1*: 37 probes and *-DRB1*: 70 probes). Subsequently, a flow analyser was used to identify the fluorescent intensity on each microsphere (LABType visual software; One lambda Inc.) and assignment of the HLA genotype was obtained on the basis of the reaction pattern.

SeCore HLA sequence-based typing was performed as follows. *HLA-A*, *HLA-B*, *HLA-C* and *HLA-DPB1* polymorphism on exon 2, exon 3 and exon 4, *HLA-DRB1* polymorphism on exon 2, and *HLA-DQB1* polymorphism on exon 2 and exon 3 were determined using the SeCore HLA Sequence-Based Typing Kit (Life Technologies Corporation) according to the manufacturer's instructions. Briefly, after locus-specific PCR amplification, PCR products were treated with exonuclease I and shrimp alkaline phosphatase to remove excess dNTP and primers. The sequencing reaction for each exon was carried out using the Big Dye terminator v1.1 cycle sequencing kit in both directions. Sequencing was run on an automated ABI 3730 sequencer (Applied Biosystems, Foster City, CA, USA). The allele assignment is obtained by comparing the determined sequence with all combinations of known allele sequence in the IMGT/HLA database using the uTYPE sequence analysis software (Life Technologies Corporation). In case of heterozygous individuals with genotype ambiguities in which several genotype combinations produced same sequencing result, allele designation was assigned according to the most common alleles found in Taiwanese population. Two-field designation with the new naming format was used to assign HLA types.

Ambiguity, which refers to the same reaction patterns produced by several genotype combinations, was dealt with by assigning allele genotypes according to common alleles (allele frequency>0.01) found in Taiwanese population and southern Chinese populations as determined in the population studies of the 13th international histocompatibility workshop.

### Genome-wide SNP genotyping and quality control

Genome-wide genotyping analysis was conducted using the Affymetrix Axiom Genome-Wide CHB 1 Array Plate with 642,832 SNPs (CapitalBio Co., Ltd, Beijing, China). Genome-wide genotypes were called by the Axiom GT1 algorithm. Systemic quality control was then performed for individuals and SNPs. For each individual genotyped in the GWAS data set, quality-control filtering for SNPs was applied to remove SNPs that were missing in >1% of samples, were not autosomal, had MAF of <1% or showed significant deviation from Hardy–Weinberg equilibrium in controls (*P*<1 × 10^−5^). We also removed SNPs for which there was a significant difference in their genotype call rates in case and control data (*P*<1 × 10^−6^). For sample filtering, arrays with generated genotypes for <95% of the loci were excluded. Heterozygosity rates were calculated, deviations of more than 6 s.d. from the mean were excluded. Heterozygosity of X-chromosome SNPs was used to verify the gender of the samples. There was no gender mismatch in our samples. The PLINK version 1.07 software (http://pngu.mgh.harvard.edu/~purcell/plink/) was used to identify samples with genetic relatedness indicating that they were from the same individual (or monozygotic twins) or from first-, second- or third-degree relatives. These determinations were based on evidence for cryptic relatedness from identity-by-descent status (pi-hat cutoff of 0.125). After filtering, we retained 522,980 autosomal SNPs in 42 TiA cases and 927 GD controls for analysis.

### Statistical analysis

We conducted MDS analysis using the PLINK version 1.07 software and principal components analysis using GCTA^38^ (http://www.complextraitgenomics.com/software/gcta/) to capture population substructure among 969 subjects. For MDS and principal components analysis, all 969 samples (42 TiA cases and 927 GD controls) were analysed together with and without the 281 Asian reference samples from the International HapMap Project (http://hapmap.ncbi.nlm.nih.gov/index.html.en/), and no outliers were identified (Supplementary Figs 3 and 4). The value of genomic inflation factor (*λ*) was 1.01, which indicated that population stratification effect was negligible in our study sample. After quality control, all GWA analyses were carried out by comparing allele/genotype frequencies between TiA cases and GD controls using five single-point methods: genotype, allele-type and Cochran–Armitage trend test along with tests considering additive effect and effective allele carrier status. The genome-wide significance threshold *P* value was set at 9.56 × 10^−8^ after Bonferroni correction for the number of SNPs (522,980). Given the excess controls in our samples, paired analyses^39^ were carried out by randomly matching 10 GD controls for each TiA case with respect to age and gender (Supplementary Table 9). Adjustments for potential genetic heterogeneity were applied by incorporating the first two or the first ten dimensions/principal components identified by MDS and GCTA^38^ (Supplementary Tables 10 and 11). The association signals at the *HLA-B* and *HLA-DRB1* regions still remained. The multivariate stepwise logistic regression analysis was performed in a stepwise (forward–backward) manner and the Cochran–Mantel–Haenszel test using SAS/STAT version 9.3 (http://www.sas.com/). The entry and removal criteria with *P* values of 0.05 based on the likelihood ratio test were used for the stepwise regression analysis. To evaluate the ability of the logistic regression model for phenotype prediction, a ROC analysis was conducted. A ROC curve to discriminate TiA cases from GD controls was generated using the combined data set, and the AUC was calculated. The LD was assessed using Haploview version 4.2 (http://www.broadinstitute.org/scientific-community/science/programs/medical-and-population-genetics/haploview/haploview). The Manhattan and quantile–quantile plots were created with the R package (http://www.r-project.org/).

### HLA imputation and association analyses

We used HLA imputation method to independently identify HLA regions from the GWAS study to validate our direct HLA typing. We imputed classical HLA alleles and corresponding amino-acid polymorphisms using Asian reference panel with high-density SNP genotypes and four-digit classical HLA allele genotypes (530 Asian individuals) using the SNP2HLA software^34^,^40^,^41^ (https://www.broadinstitute.org/mpg/snp2hla/). We extracted SNP genotypes located in the broad MHC region (chromosome 6: 29–35 Mb) to impute two-digit classical alleles, four-digit classical alleles and amino-acid polymorphisms of the eight class I and class II HLA genes (*HLA-A*, *HLA-B*, *HLA-C*, *HLA-DRB1*, *HLA-DQA1*, *HLA-DQB1*, *HLA-DPA1* and *HLA-DPB1*). PLINK was used to perform the association analyses for the HLA-imputed analysis.

### 3D model construction

The 3D structures of HLA-B*38:02 and HLA-B*38:01 were modelled by I-TASSER 4.2 (ref. 42) using the structures with PDB accession IDs of 2BCK, 3AM8, 1S7Q, 4NT6 and 1I4F as the structural templates. The HLA-DRA/DRB1*08:03 structure was constructed by MODELLER 9v9 (ref. 43) based on the structure of HLA-DR1 (PDB accession ID of 1AQD). The protonation states of the titratable residues of the proteins were assigned by PROPKA^44^ at pH 7.4 and the hydrogen atoms were added by PDB2PQR 1.9 (ref. 45). The initial conformations of the thionamide drug molecules, methimazole and propylthiouracil, were derived from Protein Data Bank with ligand IDs of MMZ and 3CJ, respectively. MarvinSketch 5.1.3 was utilized for the prediction of protonation states at pH 7.4 and the addition of hydrogen atoms to the drug molecules. Quantum chemical calculations using Gaussian03 were applied at the Hartree–Fock level with the 6-31G* basis set^46^, and the RESP (Restrained ElectroStatic Potential)^47^ scheme was employed to determine the atomic charges. Molecular dockings were performed using AutoDock4 (ref. 48), with the recalibrated scoring function (AutoDock4^RRP^)^49^. A grid box size of 18.75 × 43.5 × 18.75 Å^3^ was set to encompass the whole binding groove of the protein, and the peptide was not included to allow the drug molecules to explore all possible binding sites. Our docking results reveal that the F pocket is the most favourable pocket for drug binding (Fig. 5), with the estimated binding affinities in the range between −4.48 and −6.36 kcal mol^−1^. To further confirm the binding poses of the drug molecules, 2 ns molecular dynamics simulations were conducted for each complex system by Amberer with the AMBER parm99SB (ref. 50) for proteins and the general amber force field^51^ for drugs. The molecular graphics figures were made by PyMOL 1.3 (Fig. 4) and UCSF Chimera 1.6.1 (Fig. 5)^52^.

## Additional information

**How to cite this article:** Chen, P-L. *et al*. Genetic determinants of antithyroid drug-induced agranulocytosis by human leukocyte antigen genotyping and genome-wide association study. *Nat. Commun.* 6:7633 doi: 10.1038/ncomms8633 (2015).

## Supplementary Material

Supplementary Information Supplementary Figures 1-6 and Supplementary Tables 1-11 and Supplementary References

## Figures and Tables

**Table 1:** Association study in the first stage, second stage and combined stage.

Chr.	SNP (position*)	Study group	TiA					GD controls		
			***n***	**RAF**	**OR**†	**(95% CI)**	***P*****value**‡	***n***	**RAF**	**OR**† **(95% CI)**
6	HLA-B*38:02 (31323319)	First stage	21	0.238	7.42	(3.47–15.90)	1.59 × 10^−09^	656	0.040	1.00
		Second stage	21	0.357	21.91	(10.48–45.83)	1.59 × 10^−27^	546	0.025	1.00
		Combined stage	42	0.298	12.31	(7.33–20.67)	6.75 × 10^−32^	1202	0.033	1.00
6	rs17193122 (31335336)	First stage	21	0.262	5.74	(2.74–12.02)	8.18 × 10^−08^	474	0.058	1.00
		Second stage	21	0.381	16.77	(8.20–34.30)	1.15 × 10^−24^	453	0.035	1.00
		Combined stage	42	0.321	9.59	(5.78–15.90)	4.29 × 10^−27^	927	0.047	1.00
6	rs116869525 (32389143)	First stage	21	0.262	4.66	(2.24–9.67)	3.88 × 10^−05^	474	0.071	1.00
		Second stage	21	0.286	3.77	(1.86–7362)	8.24 × 10^−05^	453	0.096	1.00
		Combined stage	42	0.274	4.16	(2.50–6.90)	1.27 × 10^−08^	927	0.083	1.00
6	HLA-DRB1*08:03 (32552080)	First stage	21	0.286	4.76	(2.36–9.57)	1.24 × 10^−05^	651	0.078	1.00
		Second stage	21	0.286	3.96	(1.97–7.98)	3.91 × 10^−05^	545	0.092	1.00
		Combined stage	42	0.286	4.36	(2.66–7.15)	1.83 × 10^−09^	1196	0.084	1.00

Chr., chromosome; CI, confidence interval; GD, Graves' Disease; HLA, human leukocyte antigen; OR, odds ratio; RAF, risk allele frequency; TiA, thionamide-induced agranulocytosis.

^*^The physical positions were annotated according to NCBI build 37.1.

^†^OR values were calculated using allelic association test.

^‡^*P* values were calculated using Cochran–Armitage test for trend.

**Table 2:** Results of conditional association analysis using direct HLA typing and imputation.

**HLA**	**Conditional on**	**Direct HLA typing;** ***P***	**Imputed HLA using SNP2HLA;** P*
HLA-B*38:02	HLA-DRB1*08:03	1.89 × 10^−16^	2.07 × 10^−14^
HLA-DRB1*08:03	HLA-B*38:02	2.24 × 10^−6^	1.68 × 10^−6^

HLA, human leukocyte antigen; SNP, single-nucleotide polymorphism.

^*^SNP2HLA (Jia *et al*.^34^,^40^,^41^)
