# Supplementary material for: Genetic determinants of antithyroid drug-induced agranulocytosis by human leukocyte antigen genotyping and genome-wide association study
Source: Nat Commun. 2015 Jul 7;6:7633. doi: 10.1038/ncomms8633 (PMC4506516; doi:10.1038/ncomms8633)
Supplement: Supplementary Information — Supplementary Figures 1-6 and Supplementary Tables 1-11 and Supplementary References [file ncomms8633-s1.pdf]

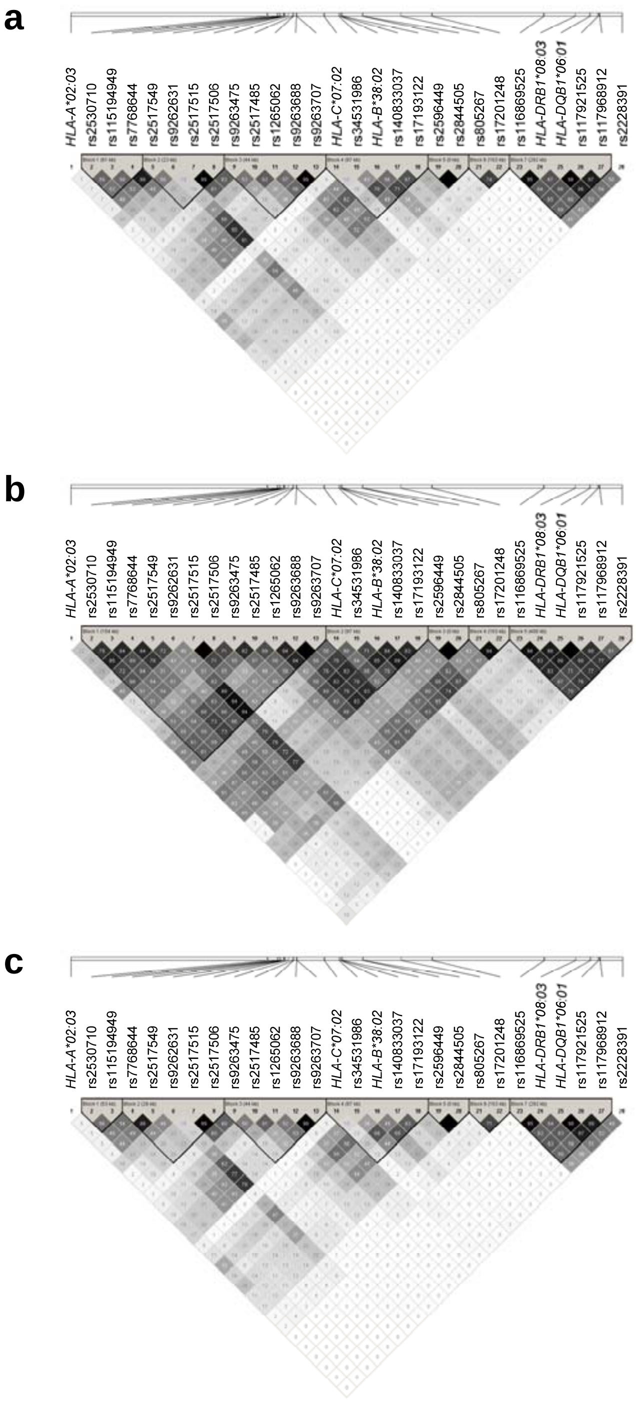

Supplementary Figure 1 The linkage disequilibrium plots of 5 selected HLA genes/alleles and 23 SNPs at the HLA regions. (**a**) in all combined 42 TiA cases and 1,208 GD controls, (**b**) in 42 TiA cases, and (**c**) in 1,208 GD controls. The numbers inside individual cells are the r^2 values (× 100%) of corresponding allele pairs.

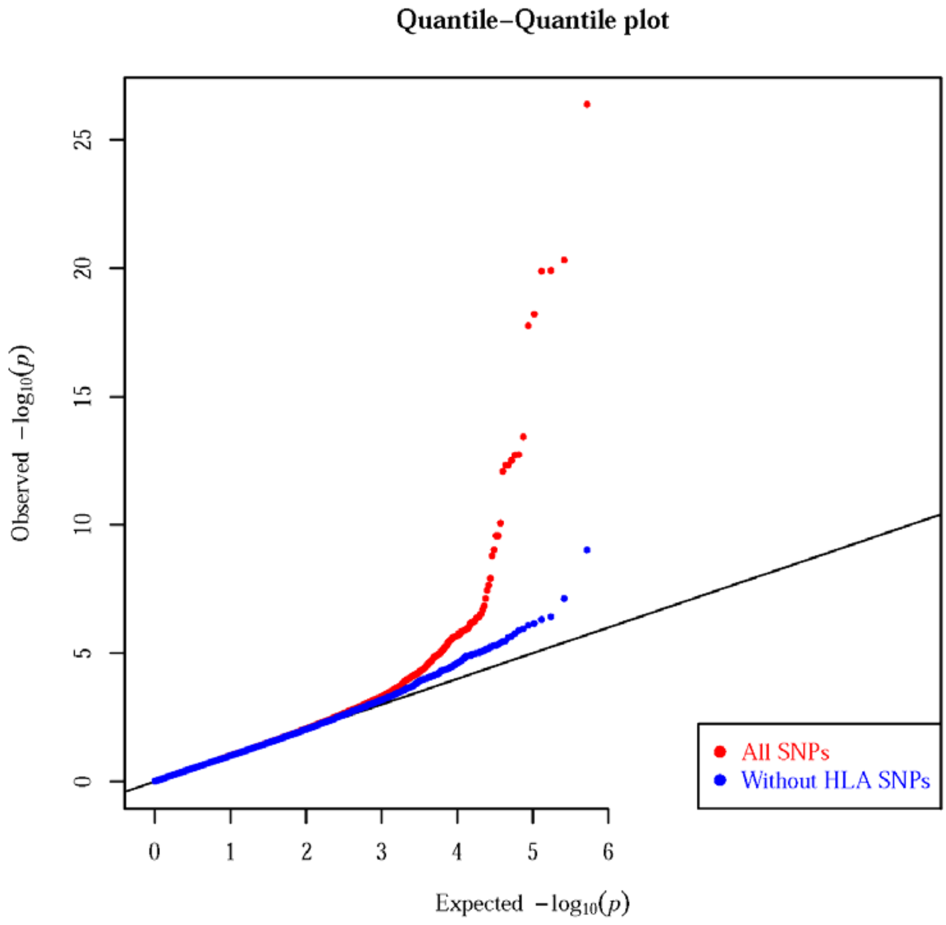

Supplementary Figure 2 Quantile-quantile plot (Q-Q plot) for test statistic in 42 TiA cases compared with 927 GD controls.

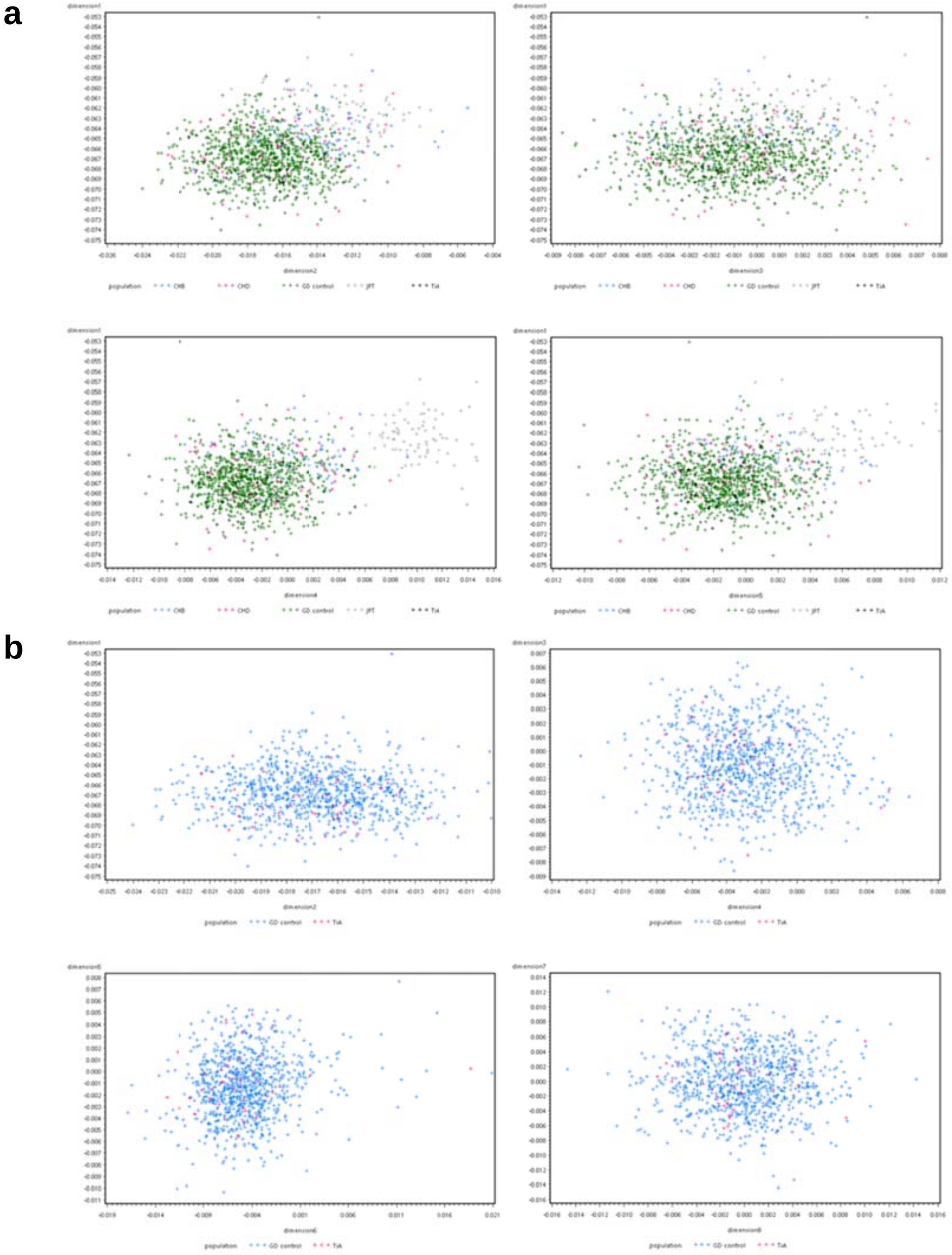

Supplementary Figure 3 The multidimensional scaling (MDS) plot. (**a**) The MDS plot of 969 GWAS samples (denoted as “TiA” for the 42 TiA cases and “GD control” for 927 GD controls) and 281 ASN reference DNA samples (CHB+CHD+JPT) from HapMap. (**b**) Plots of the first eight dimensions from the MDS analysis using 969 samples (42 TiA and 927 GD controls).

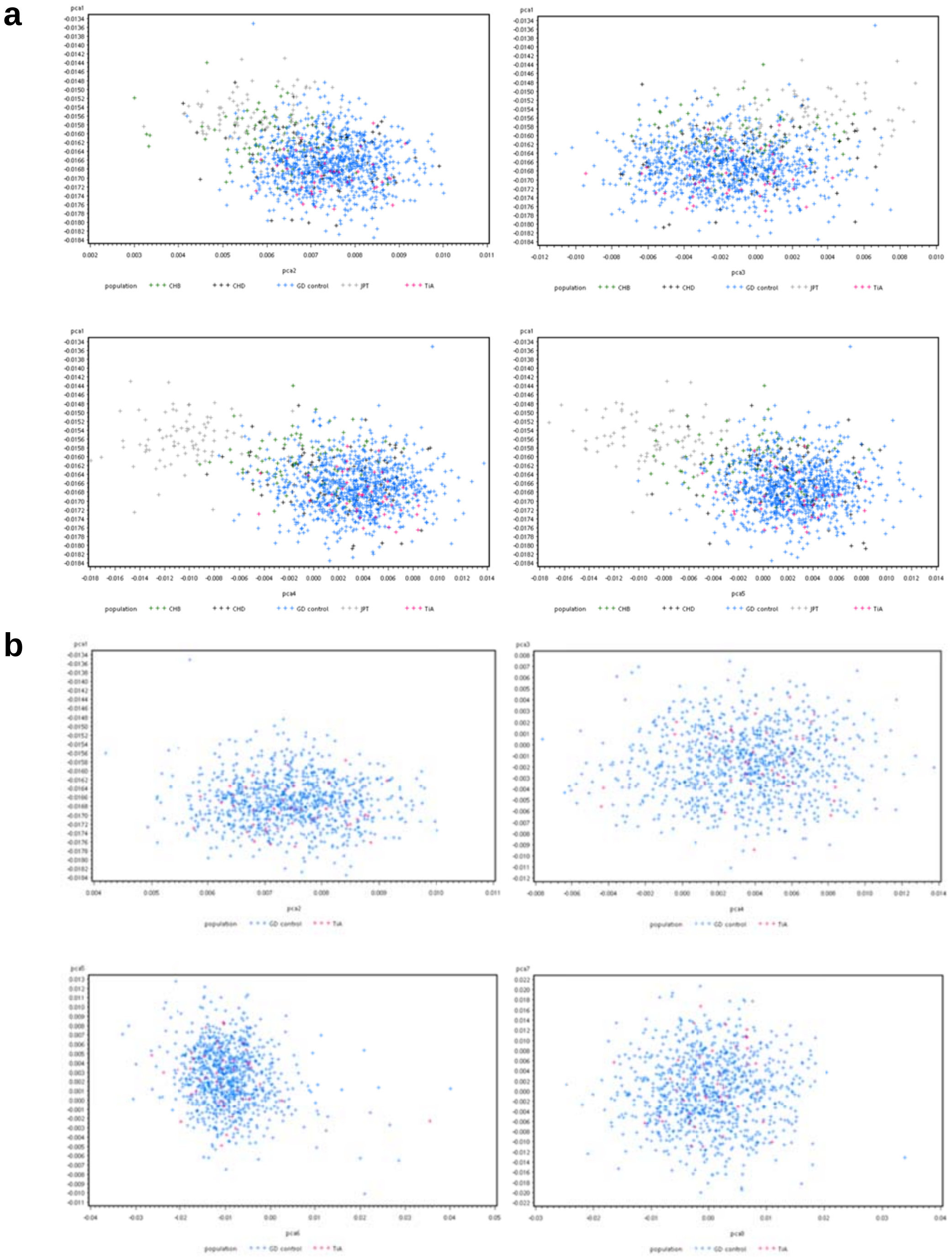

Supplementary Figure 4 The principal-component analysis (PCA). (**a**) PCA of 969 samples and 281 ASN reference DNA samples (CHB+CHD+JPT) from HapMap. (**b**) Plots of first eight principal-components from the PCA using 969 samples (42 TiA cases and 927 GD controls).

```
AA Position         -21        -11         -1         10         20         30         40         50         60         70
B*38:02:01         MLVM APRTVLLLLS AALALTETWA GSHSMRYFYT SVSRPGRGEP RFISVGYVDD TQFVRFDSDA ASPREEPRAP WIEQEGPEYW DRNTQICKTN
B*38:01:01         ---- ---------- ---------- ---------- ---------- ---------- ---------- ---------- ---------- ----------
B*38:15            **** ********** ********** *--------- ---------- ---------- ---------- ---------- ---------- ----------
AA Position          80         90        100        110        120        130        140        150        160        170
B*38:02:01   TQTYRENLRT ALRYYNQSEA GSHTLQRMYG CDVGPDGRLL RGHNQFAYDG KDYIALNEDL SSWTAADTAA QITQRKWEAA RVAEQLRTYL EGTCVEWLRR
B*38:01:01   ---------I ---------- ---------- ---------- ---------- ---------- ---------- ---------- ---------- ----------
B*38:15      ---------- ---------- -P-------- ---------- ---------- ---------- ---------- ---------- ---------- ----------
AA Position         180        190        200        210        220        230        240        250        260        270
B*38:02:01   YLENGKETLQ RADPPKTHVT HHPISDHEAT LRCWALGFYP AEITLTWQRD GEDQTQDTEL VETRPAGDRT FQKWAAVVVP SGEEQRYTCH VQHEGLPKPL
B*38:01:01   ---------- ---------- ---------- ---------- ---------- ---------- ---------- ---------- ---------- ----------
B*38:15      ---------- --******** ********** ********** ********** ********** ********** ********** ********** **********
AA Position         280        290         300        310        320        330
B*38:02:01   TLRWEPSSQS TVPIVGIVAG LAVL.AVVVIG AVVAAVMCRR KSSGGKGGSY SQAASSDSAQ GSDVSLTA
B*38:01:01   ---------- ---------- ----.------ ---------- ---------- ---------- --------
B*38:15      ********** ********** *********** ********** ********** ********** ********
```

Supplementary Figure 5 Amino acid sequence alignment of *HLA-B*38:02*, *HLA-B*38:01* and *HLA-B*38:15*.

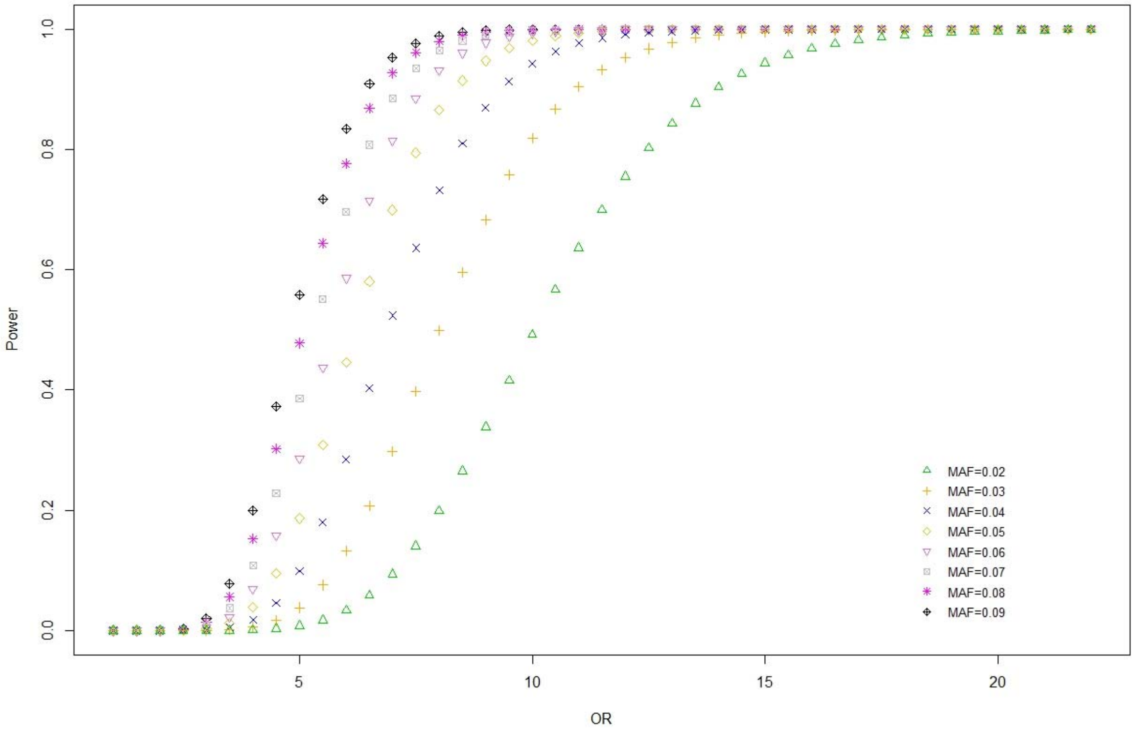

Supplementary Figure 6 Power calculation. The statistic power was calculated with sample size of the current GWAS study (TiA case number = 42, GD control number = 927) under various minor allele frequencies (MAFs) and odds ratios (ORs) settings using QUANTO[1]. A cut-off *P* value of 1×10^{-8} was used for the calculation.

Supplementary Table 1 Study design and number of genotyped participants

Study Group	Sample Group	N	Genotyping	Enrolled time
First-stage	TiA cases	21	Axiom CHB GWAS SNP genotyping<br>Direct HLA genotyping	2001-2012
	GD controls<br>(Unrelated Singletons)	497	Axiom CHB GWAS SNP genotyping<br>Direct HLA genotyping	2001-2006
	GD controls<br>(Unrelated Family Probands)	165	Direct HLA genotyping	2001-2006
Second-stage	TiA cases	21	Axiom CHB GWAS SNP genotyping<br>Direct HLA genotyping	2013-2014
	GD controls<br>(Unrelated Singletons)	546	Axiom CHB GWAS SNP genotyping<br>Direct HLA genotyping	2001-2011

Supplementary Table 2 Genotypic information of 42 TiA cases

Study group	Study subject	Genotype of *HLA-B*	Genotype of rs17193122	Genotype of rs116869525	Genotype of *HLA-DRB1*
First-stage	Stage1_01	48:01/55:02	GG	CC	04:05/15:01
	Stage1_02	27:04/46:01	GG	CC	04:05/09:01
	Stage1_03	38:02/46:01	AG	TC	08:03/09:01
	Stage1_04	38:02/46:01	AG	TT	08:03/08:03
	Stage1_05	38:02/46:01	AG	CC	09:01/09:01
	Stage1_06	15:02/39:01	GG	TC	08:03/16:02
	Stage1_07	38:02/40:01	AG	TC	08:03/16:02
	Stage1_08	46:01/48:03	GG	CC	09:01/12:02
	Stage1_09	38:02/46:01	AG	CC	04:03/04:06
	Stage1_10	35:01/38:02	AG	CC	15:01/15:02
	Stage1_11	15:11/38:02	AG	TC	08:03/15:01
	Stage1_12	40:01/54:01	GG	CC	11:01/14:05
	Stage1_13	46:01/58:01	GG	CC	03:01/04:05
	Stage1_14	46:01/52:01	GG	CC	07:01/15:01
	Stage1_15	13:02/51:02	GG	CC	04:05/07:01
	Stage1_16	38:02/51:01	AG	TC	08:03/09:01
	Stage1_17	38:15/40:01	AG	TT	08:03/08:03
	Stage1_18	35:03/38:02	AG	CC	08:03/16:02
	Stage1_19	40:01/58:01	GG	CC	09:01/09:01
	Stage1_20	15:01/15:01	GG	TC	08:03/09:01
	Stage1_21	27:04/38:02	AG	TC	04:06/08:03
	Stage2_01	37:01/38:02	AG	TC	08:03/10:01
	Stage2_02	38:02/40:02	AG	CC	15:01/16:02
	Stage2_03	40:06/51:01	GG	CC	09:01/12:01
	Stage2_04	40:01/46:01	GG	TC	08:03/08:09
	Stage2_05	38:02/40:01	AG	CC	12:01/16:02
	Stage2_06	38:02/51:01	AG	TC	08:03/16:02
	Stage2_07	38:02/46:01	AG	CC	09:01/16:02

Second-stage	Stage2_08	13:01/38:02	AG	CC	15:01/15:02
	Stage2_09	38:02/40:01	AG	CC	11:01/11:01
	Stage2_10	38:02/46:01	AG	TC	08:03/12:01
	Stage2_11	38:02/46:01	AG	TC	08:03/09:01
	Stage2_12	38:02/40:01	AG	TC	04:03/08:03
	Stage2_13	39:01/54:01	GG	TC	08:03/09:01
	Stage2_14	38:02/51:02	AG	TC	08:03/16:02
	Stage2_15	35:05/46:01	GG	CC	04:05/12:02
	Stage2_16	58:01/58:01	GG	CC	03:01/03:01
	Stage2_17	13:01/54:01	AG	TC	08:03/15:01
	Stage2_18	38:02/40:01	AG	TC	08:03/09:01
	Stage2_19	38:02/46:01	AG	CC	04:06/09:01
	Stage2_20	38:02/51:01	AG	TC	04:06/08:03
	Stage2_21	38:02/51:02	AG	TC	08:03/15:02

Supplementary Table 3 Results of association analysis using direct HLA typing and SNP imputation

HLA	Position	Direct HLA typing				Imputed HLA using SNP2HLA[a]			
		GD control MAF	TiA MAF	OR (95% CI)	$P_{additive}$	GD control MAF	TiA MAF	OR (95% CI)	$P_{additive}$
*HLA-B*38:02*	31323319	0.0333	0.2976	14.89 (7.98-27.78)	8.35×10^{-17}	0.0432	0.3095	12.57 (6.73-23.46)	1.87×10^{-15}
*HLA-DRB1*08:03*	32552080	0.0840	0.2857	3.68 (2.30-5.90)	1.33×10^{-7}	0.0868	0.2976	3.93 (2.42-6.38)	3.26×10^{-8}

MAF, minor allele frequency; OR, odds ratio.
[a]SNP2HLA[2-4]

Supplementary Table 4 Association results of 24 SNPs in the combined association study of 42 TiA cases and 927 GD controls

dbSNP_ID	Chr.	Position[a]	Allele[b]	MAF		Additive		Allelic		Trend
				TiA (%)	GD control (%)	OR (95% CI)	*P*	OR (95% CI)	*P*	*P*
rs56343172	3	111587245	T/C	14.3	3.1	5.47 (2.71-11.05)	2.12E-06	5.15 (2.65-10.01)	8.72E-08	7.75E-08
rs2530710	6	30940387	T/C	38.1	11.6	5.11 (3.08-8.49)	2.94E-10	4.70 (2.96-7.46)	1.00E-12	8.55E-13
rs115194949	6	30993440	A/G	35.7	10.1	5.18 (3.11-8.63)	2.56E-10	4.92 (3.07-7.87)	4.00E-13	4.90E-13
rs7768644	6	31002101	A/G	33.3	9.1	5.78 (3.35-9.95)	2.66E-10	5.01 (3.10-8.10)	5.86E-13	3.24E-13
rs2517549	6	31008598	A/C	29.8	8.7	5.00 (2.89-8.64)	8.39E-09	4.45 (2.71-7.30)	1.47E-10	8.94E-11
rs9262631	6	31024601	A/C	32.1	6.3	8.39 (4.70-14.98)	6.79E-13	7.09 (4.32-11.63)	7.34E-19	6.19E-19
rs2517515	6	31029258	T/C	48.8	27.1	2.90 (1.84-4.56)	4.12E-06	2.83 (1.82-4.39)	1.52E-06	1.38E-06
rs2517506	6	31031680	C/T	48.8	27.1	2.89 (1.84-4.55)	4.29E-06	2.82 (1.81-4.73)	1.63E-06	1.45E-06
rs9263475	6	31050348	G/A	48.8	23.5	3.06 (1.96-4.78)	9.28E-07	3.10 (1.99-4.81)	1.47E-07	2.04E-07
rs2517485	6	31074101	T/C	44.1	13.7	4.24 (2.73-6.58)	1.41E-10	4.95 (3.15-7.76)	2.93E-14	4.91E-13
rs1265062	6	31078005	T/C	34.5	8.8	5.33 (3.22-8.82)	7.13E-11	5.47 (3.39-8.82)	1.15E-14	3.74E-14
rs9263688	6	31091967	G/A	31.0	5.3	9.62 (5.29-17.48)	1.14E-13	8.06 (4.86-13.37)	4.69E-21	4.96E-21
rs9263707	6	31095246	T/C	31.0	5.3	8.91 (4.98-15.96)	1.91E-13	8.02 (4.84-13.30)	5.73E-21	1.25E-20
rs34531986	6	31315587	T/G	36.6	8.2	8.51 (4.72-15.32)	9.70E-13	6.48 (4.02-10.47)	5.79E-18	1.78E-18
rs140833037	6	31325056	T/C	33.3	6.3	9.70 (5.32-17.69)	1.24E-13	7.42 (4.54-12.13)	3.35E-20	1.32E-20
rs17193122	6	31335336	A/G	32.1	4.7	17.32 (8.87-33.80)	6.34E-17	9.59 (5.78-15.9)	1.69E-25	4.29E-27
rs2596449	6	31438390	A/G	23.8	4.7	6.14 (3.45-10.94)	7.24E-10	6.35 (3.68-10.96)	6.22E-14	1.90E-13
rs2844505	6	31439063	C/T	23.8	4.7	6.13 (3.44-10.93)	7.39E-10	6.34 (3.67-10.95)	6.50E-14	1.98E-13
rs805267	6	31639757	A/G	27.4	10.4	3.62 (2.10-6.24)	3.79E-06	3.26 (1.98-5.39)	1.18E-06	6.96E-07
rs17201248	6	31803130	T/C	26.2	9.2	3.71 (2.16-6.39)	2.22E-06	3.48 (2.09-5.81)	4.01E-07	3.11E-07
rs116869525	6	32389143	T/C	27.4	8.3	3.66 (2.24-6.00)	2.49E-07	4.16 (2.50-6.90)	3.03E-09	1.27E-08
rs117921525	6	32672734	A/C	31.0	12.3	3.03 (1.88-4.89)	5.55E-06	3.20 (1.98-5.19)	7.03E-07	1.28E-06
rs117968912	6	32681179	T/G	35.7	13.2	3.34 (2.11-5.27)	2.47E-07	3.65 (2.29-5.82)	7.47E-09	2.37E-08
rs2228391	6	32797773	C/T	28.6	10.8	3.52 (2.08-5.95)	2.57E-06	3.32 (2.02-5.46)	5.57E-07	4.35E-07

Chr., chromosome; MAF, minor allele frequency; OR, odds ratio; CI, confidence interval.
[a]Position according to the 19th version of the human reference genome from the Genome Reference Consortium (hg19). [b]The first allele indicates the risk allele, and the reference allele is listed in second position.

Supplementary Table 5 HLA alleles selected by the stepwise logistic regression analysis

SNP	OR (95% CI)	Z-value	Stepwise regression: *P* value in final model
*HLA-B*38:02*	13.99 (7.46-26.22)	8.23	1.89×10^{-16}
*HLA-DRB1*08:03*	3.62 (2.12-6.16)	4.73	2.24×10^{-6}

OR, odds ratio; CI, confidence interval.

Supplementary Table 6 Baseline case characteristics between the two HLA genotype groups

Characteristics	In 42 TiA cases				
	Without ***B*38:02*** or ***DRB1*08:03*** (n = 11)	***B*38:02*** only (n = 9)	***DRB1*08:03*** only (n = 6)	Both ***B*38:02*** and ***DRB1*08:03*** (n = 16)	***P***
Female sex – no (%)[a]	10 (90.91)	7 (77.78)	5 (83.33)	15 (93.75)	0.623
Age – year[b]	53 (29-77)	53 (25-66)	34 (21-51)	50 (25-66)	0.131
Age at agranulocytosis – year[b]	41 (18-67)	42 (22-57)	28 (20-33)	40 (17-50)	0.026
Onset of neutropenia – day[b]	30 (3-180)	43.5 (28-66)	30 (12-60)	34 (12-60)	0.643

[a]*P* value was calculated using Fisher's exact test. [b]Values were presented as: median (range). *P* values for continuous variables were calculated using the Kruskal-Wallis Test.

Supplementary Table 7 Relationship between HLA genotypes and time from starting the drug to development of neutropenia

**Characteristics**	***HLA-B*38:02***			***HLA-DRB1*08:03***			**Both *B*38:02* and *DRB1*08:03***		
	Carrier (n = 22)	Non-carrier (n = 15)	*P*	Carrier (n = 19)	Non-carrier (n = 18)	*P*	Carrier (n = 14)	Non-carrier (n = 23)	*P*
Onset of neutropenia - day[a]	39 (12-66)	30 (3-180)	0.527	30 (12-60)	30 (3-180)	0.496	34 (12-60)	30 (3-180)	0.684

[a]Values were presented as: median (range).

Supplementary Table 8 Associations between HLA genotypes and medications

HLA type	Study group (no. of samples)	Genotype Frequency Number (percent)			(Homozygous+Heterozygous) odds ratio (PP^a+PA^a vs. AA^a)		Allelic odds ratio (P^a vs. A^a)	
		Homozygous (PP^a)	Heterozygous (PA^a)	Noncarrier (AA^a)	OR (95% CI)	*P* value[b]	OR (95% CI)	*P* value[b]
HLA-B*38:02	**TiA (42)**	0 (0)	25 (59.52)	17 (40.48)	21.49 (11.13-41.49)	3.14E-18	12.31 (7.33-20.67)	2.28E-32
	Carbimazole (9)	0 (0)	3 (33.33)	6 (66.67)	7.31 (1.79-29.77)	1.75E-2	5.81 (1.65-20.47)	1.90E-3
	Methimazole (23)	0 (0)	18 (78.26)	5 (21.74)	52.60 (19.02-145.48)	5.15E-17	18.68 (9.92-35.16)	1.24E-34
	Propylthiouracil (9)	0 (0)	3 (33.33)	6 (66.67)	7.31 (1.79-29.77)	1.75E-2	5.81 (1.65-20.47)	1.90E-3
	**Controls[c] (1208)**	3 (0.25)	74 (6.16)	1125 (93.59)	1.00		1.00	
HLA-DRB1*08:03	**TiA (42)**	2 (4.76)	20 (47.62)	20 (47.62)	6.13 (3.28-11.46)	5.08E-8	4.36 (2.66-7.15)	2.60E-10
	Carbimazole (9)	0 (0)	5 (55.56)	4 (44.44)	6.96 (1.85-26.18)	6.30E-3	4.19 (1.48-11.88)	3.40E-3
	Methimazole (23)	2 (8.70)	12 (52.17)	9 (39.13)	8.67 (3.70-20.32)	1.08E-6	5.81 (3.12-10.85)	4.86E-10
	Propylthiouracil (9)	0 (0)	2 (22.22)	7 (77.78)	1.59 (0.33-7.72)	6.34E-1	1.36 (0.31-5.97)	6.80E-1
	**Controls[c] (1208)**	19 (1.59)	163 (13.63)	1014 (84.78)	1.00		1.00	

[a]P, present; A, absent. [b]*P* values were derived from comparison with overall distribution in GD controls. [c]According to the Taiwan National Health Insurance Research Database (NHIRD), the proportions of GD patients prescribed with carbimazole, methimazole and propylthiouracil were 0.2, 0.485 and 0.315, respectively (unpublished data), which were not statistically different ($P = 0.40$) from those for the TiA patients (0.22, 0.56, and 0.22).

Supplementary Table 9 Results of association analysis using Cochran-Armitage trend test with 1:10 age- and gender-matched cases and controls in the combined samples

SNPs	Chr.	Allele 1	Allele 2	Combined samples	
				Original	1:10 matched for age and gender
				P_{trend}	Median[a] of P_{trend}
rs56343172	3	T	C	7.75E-08	1.67E-06
rs2530710	6	T	C	8.55E-13	2.17E-11
rs115194949	6	A	G	4.90E-13	1.31E-11
rs7768644	6	A	G	3.24E-13	5.76E-12
rs2517549	6	A	C	8.94E-11	7.18E-10
rs9262631	6	A	C	6.19E-19	2.54E-16
rs2517515	6	T	C	1.38E-06	2.70E-06
rs2517506	6	C	T	1.45E-06	2.88E-06
rs9263475	6	G	A	2.04E-07	5.23E-07
rs2517485	6	T	C	4.91E-13	1.19E-11
rs1265062	6	T	C	3.74E-14	7.25E-13
rs9263688	6	G	A	4.96E-21	8.73E-18
rs9263707	6	T	C	1.25E-20	1.07E-17
rs34531986	6	T	G	1.78E-18	9.27E-16
rs140833037	6	T	C	1.32E-20	2.10E-17
rs17193122	6	A	G	4.29E-27	3.51E-22
rs2596449	6	A	G	1.90E-13	9.96E-11
rs2844505	6	C	T	1.98E-13	9.96E-11
rs805267	6	A	G	6.96E-07	2.37E-06
rs17201248	6	T	C	3.11E-07	1.73E-06
rs116869525	6	T	C	1.27E-08	2.37E-07
rs117921525	6	A	C	1.28E-06	1.08E-05
rs117968912	6	T	G	2.37E-08	3.96E-07
rs2228391	6	C	T	4.35E-07	3.34E-06

Chr., chromosome.
[a]1,000 bootstraps.

Supplementary Table 10 Results of association analysis using Cochran-Armitage trend test adjusting for potential population substructure by the first two and the first 10 dimensions from multidimensional scaling (MDS) analysis in the combined samples.

SNPs	Chr.	Allele 1	Allele 2	Combined samples		
				Original	Adjusted	
				P_{trend}	P^{a}_{trend}	P^{b}_{trend}
rs56343172	3	T	C	7.75E-08	1.15E-06	5.61E-07
rs2530710	6	T	C	8.55E-13	6.90E-10	8.20E-10
rs115194949	6	A	G	4.90E-13	5.84E-10	6.41E-10
rs7768644	6	A	G	3.24E-13	5.03E-10	3.39E-10
rs2517549	6	A	C	8.94E-11	1.54E-08	7.40E-09
rs9262631	6	A	C	6.19E-19	1.57E-12	3.56E-13
rs2517515	6	T	C	1.38E-06	4.86E-06	1.71E-06
rs2517506	6	C	T	1.45E-06	4.99E-06	1.76E-06
rs9263475	6	G	A	2.04E-07	1.22E-06	2.58E-07
rs2517485	6	T	C	4.91E-13	2.70E-10	1.58E-11
rs1265062	6	T	C	3.74E-14	1.36E-10	3.33E-11
rs9263688	6	G	A	4.96E-21	4.18E-13	1.39E-13
rs9263707	6	T	C	1.25E-20	5.47E-13	2.06E-13
rs34531986	6	T	G	1.78E-18	7.95E-13	3.68E-13
rs140833037	6	T	C	1.32E-20	6.38E-14	3.25E-14
rs17193122	6	A	G	4.29E-27	1.23E-16	1.47E-16
rs2596449	6	A	G	1.90E-13	5.00E-10	8.28E-10
rs2844505	6	C	T	1.98E-13	5.09E-10	8.42E-10
rs805267	6	A	G	6.96E-07	2.80E-06	3.35E-06
rs17201248	6	T	C	3.11E-07	1.01E-06	1.87E-06
rs116869525	6	T	C	1.27E-08	5.60E-07	9.28E-07
rs117921525	6	A	C	1.28E-06	1.09E-05	2.15E-05
rs117968912	6	T	G	2.37E-08	5.99E-07	1.45E-06
rs2228391	6	C	T	4.35E-07	2.39E-06	2.93E-06

Chr., chromosome.
[a]Adjusted for the first two dimensions. [b]Adjusted for the first 10 dimensions.

Supplementary Table 11 Results of association analysis using Cochran-Armitage trend test adjusting for the first two and the first 10 principal components identified by GCTA in the combined samples

SNPs	Chr.	A1	A2	Combined samples		
				Original	Adjusted	
				P_{trend}	P^{a}_{trend}	P^{b}_{trend}
rs56343172	3	T	C	7.75E-08	1.15E-06	4.97E-07
rs2530710	6	T	C	8.55E-13	6.20E-10	6.35E-10
rs115194949	6	A	G	4.90E-13	5.08E-10	4.24E-10
rs7768644	6	A	G	3.24E-13	4.51E-10	2.81E-10
rs2517549	6	A	C	8.94E-11	1.46E-08	8.44E-09
rs9262631	6	A	C	6.19E-19	1.72E-12	5.52E-13
rs2517515	6	T	C	1.38E-06	5.11E-06	2.15E-06
rs2517506	6	C	T	1.45E-06	5.27E-06	2.22E-06
rs9263475	6	G	A	2.04E-07	1.34E-06	3.79E-07
rs2517485	6	T	C	4.91E-13	2.93E-10	3.13E-11
rs1265062	6	T	C	3.74E-14	1.38E-10	6.47E-11
rs9263688	6	G	A	4.96E-21	3.67E-13	2.11E-13
rs9263707	6	T	C	1.25E-20	5.54E-13	3.65E-13
rs34531986	6	T	G	1.78E-18	8.15E-13	5.72E-13
rs140833037	6	T	C	1.32E-20	5.92E-14	4.96E-14
rs17193122	6	A	G	4.29E-27	1.06E-16	1.55E-16
rs2596449	6	A	G	1.90E-13	4.82E-10	9.50E-10
rs2844505	6	C	T	1.98E-13	4.90E-10	9.70E-10
rs805267	6	A	G	6.96E-07	2.61E-06	3.57E-06
rs17201248	6	T	C	3.11E-07	1.06E-06	1.72E-06
rs116869525	6	T	C	1.27E-08	4.26E-07	1.02E-06
rs117921525	6	A	C	1.28E-06	8.84E-06	2.07E-05
rs117968912	6	T	G	2.37E-08	4.63E-07	1.36E-06
rs2228391	6	C	T	4.35E-07	2.30E-06	3.94E-06

GCTA, genome-wide complex trait analysis (Yang *et al.*[5]); Chr., chromosome.
[a]Adjusted for the first 2 principal components. [b]Adjusted for the first 10 principal components.

Supplementary references

1. Gauderman, W.J. Sample size requirements for association studies of gene-gene interaction. *Am. J. Epidemiol.* **155**, 478-484 (2002).
2. Okada, Y. *et al.* Risk for ACPA-positive rheumatoid arthritis is driven by shared HLA amino acid polymorphisms in Asian and European populations. *Hum. Mol. Genet.* **23**, 6916-6926 (2014).
3. Jia, X. *et al.* Imputing amino acid polymorphisms in human leukocyte antigens. *PLoS One* **8**, e64683 (2013).
4. Pillai, N.E. *et al.* Predicting HLA alleles from high-resolution SNP data in three Southeast Asian populations. *Hum. Mol. Genet.* **23**, 4443-4451 (2014).
5. Yang, J., Lee, S.H., Goddard, M.E. & Visscher, P.M. GCTA: a tool for genome-wide complex trait analysis. *Am. J. Hum. Genet.* **88**, 76-82 (2011).
